# The mutational landscape and its longitudinal dynamics in relapsed and refractory classic Hodgkin lymphoma

**DOI:** 10.1007/s00277-025-06274-5

**Published:** 2025-02-24

**Authors:** Hanno Witte, Axel Künstner, Thomas Hahn, Veronica Bernard, Stephanie Stölting, Kathrin Kusch, Kumar Nagarathinam, Cyrus Khandanpour, Nikolas von Bubnoff, Arthur Bauer, Michael Grunert, Svenja Hartung, Annette Arndt, Konrad Steinestel, Hartmut Merz, Hauke Busch, Alfred C. Feller, Niklas Gebauer

**Affiliations:** 1 University Cancer Center Schleswig-Holstein, University Hospital of Schleswig-Holstein, Campus Lübeck, 23538 Lübeck, Germany; 2 Department of Hematology and Oncology, Bundeswehrkrankenhaus Ulm, Oberer Eselsberg 40, 89081 Ulm, Germany; 3 Department of Hematology and Oncology, University Hospital of Schleswig-Holstein, Campus Lübeck, Ratzeburger Allee 160, 23538 Lübeck, Germany; 4 Medical Systems Biology Group, University of Lübeck, Ratzeburger Allee 160, 23538 Lübeck, Germany; 5 Institute for Cardiogenetics, University of Lübeck, Ratzeburger Allee 160, 23538 Lübeck, Germany; 6 Hämatopathologie Lübeck, Reference Centre for Lymph Node Pathology and Hematopathology, Maria-Goeppert-Straße 9a, 23562 Lübeck, Germany; 7 Institute of Biochemistry, University of Lübeck, Ratzeburger Allee 160, 23538 Lübeck, Germany; 8 Department of Nuclear Medicine, Bundeswehrkrankenhaus Ulm, Oberer Eselsberg 40, 89081 Ulm, Germany; 9 Institute of Pathology, University Ulm, Albert-Einstein Allee 23, 89081 Ulm, Germany; 10 Institute of Pathology and Molecularpathology, Bundeswehrkrankenhaus Ulm, Oberer Eselsberg 40, 89081 Ulm, Germany

**Keywords:** Relapsed/refractory classic Hodgkin lymphoma, Ultra-deep sequencing, Mutational landscape, Clonal evolution

## Abstract

In classic Hodgkin-lymphoma (cHL), only a few cases recur, and only a limited fraction of patients is primary-refractory to standard-polychemotherapy. Underlying genomic features of unfavorable clinical courses remain sparsely characterized. Here, we investigated the genomic characteristics of primary-refractory/relapsed cHL in contrast with responders. Therefore, ultra-deep next-generation panel-sequencing was performed on a total of 59 FFPE-samples (20 responders, 26 relapsed (rHL: 11 initial-diagnosis, 15 relapse) and 13 primary-refractory (prHL: 8 initial-diagnosis, 5 progression) from 44 cHL-patients applying a hybrid-capture approach. We compared samples associated with distinct disease courses concerning their oncogenic drivers, mutational signatures, and perturbed pathways. Compared to responders, mutations in genes such as *PMS2, PDGFRB, KAT6A, EPHB1,* and *HGF* were detected more frequently in prHL/rHL. Additionally, we observed that in rHL or prHL, *BARD1-*mutations occur, whereas *ETV1, NF1,* and *MET*-mutations were eliminated through clonal selection. A significant enrichment of non-synonymous variants was detected in prHL compared to responders and a significant selection process in favor of NOTCH-pathway mutations driving rHL or prHL was observed. However, our analysis revealed a negative selection process for non-synonymous variants affecting the hippo-pathway. This study delineates distinct mutational signatures between responders and rHL/prHL, whilst illustrating longitudinal dynamics in mutational profiles using paired samples. Further, several exploitable therapeutic vulnerabilities for rHL and prHL were identified.

**Supplementary Information:**

The online version contains supplementary material available at 10.1007/s00277-025-06274-5.

## Introduction

Classic Hodgkin lymphoma (cHL) is a malignant lymphoma of B-cell lineage that is highly curable with intensive and oftentimes positron-emission-tomography (PET)-guided intensification of chemotherapy and/or radiotherapy throughout all stages [1, 2]. In elderly and/or relapsed/refractory patients there is a need for treatment individualization to enable targeted and less toxic therapeutic approaches and to overcome resistant disease. Further, de-escalation of frontline therapy is a central aspect in the effort to reduce early- and late toxicities including cardiovascular disease, infertility, fatigue, and secondary cancers as main causes of morbidity and mortality in this population [3]. New therapy standards considering such factors have recently been set for patients with advanced stage disease [4, 5].

Several previously published studies have outlined the pathogenetic key functions of the NF $\kappa$ B, PI3K/AKT/mTOR, and IL6/JAK/STAT pathways in lymphomagenesis of cHL [6–11]. Moreover, distinct pathogenetic features of EBV-positive versus EBV-negative cHL were recently introduced [12]. In their pivotal study, Sobesky et al*.* recently depicted the genomic landscape of cHL employing a platform for deep cell-free DNA (cfDNA) sequencing and outlined its applicability for sensitive detection of minimal residual disease [13]. Despite the overall favourable prognosis, primary-refractory disease and relapse events still occur. Such cases represent a major challenge for treating physicians. In one study, 12 primary-refractory cHL cases were investigated using a panel-sequencing approach of 35 candidate driver genes. Here, frequent *TP53* mutations as well as mutations affecting epigenetic regulators were detected [14]. To date, less is known about the underlying genomic mechanisms of such unfavourable cases and the longitudinal dynamics of genetic alterations to predict primary-refractory disease or relapse events early enough. There exists an unmet medical need for the establishment of biomarkers to guide individualized precision oncology approaches in cHL beyond PET-based treatment intensification. Accordingly, a complementary molecular analysis of both early responders and refractory cases as well as cases developing subsequent relapses is required.

In this retrospective study, we sought to investigate the mutational landscape in cHL with an emphasis on the molecular trajectories in relapsed/refractory patients by ultra-deep hybrid capture sequencing.

## Methods

### Sample acquisition and clinicopathological characteristics

In this retrospective study, patients with cHL from the participating departments for Hematology and Oncology of the University Hospital Schleswig–Holstein (UKSH Campus Lübeck) and the Sana Hospital Lübeck that received systemic polychemotherapy between January 2002 and December 2022 were screened about their inclusion. The initial screening procedure was limited to cHL cases undergoing first line treatment. However, for this study the screening procedure was expanded focusing on cHL cases that experienced relapse or primary-refractory disease. HIV-positive cHL cases were excluded from this study. From 44 cases meeting diagnostic and clinical criteria for cHL, we included 8 cases of primary refractory (prHL) disease (sequential samples from initial diagnosis and primary refractory setting were available in 5 cases; in three primary refractory cases only samples from initial diagnosis were available), 16 cases of relapsed (rHL) disease (sequential samples from initial diagnosis and the timepoint of clinical relapse were available in 10 cases) and 20 responders towards polychemotherapy for targeted sequencing. Responders were defined as cHL cases responding to first-line standard polychemotherapy without experiencing any relapse event with a follow-up period of at least 12 months after treatment completion. Of the initially reviewed 84 samples 25 specimens were dismissed from analysis due to quality control issues following library preparation. The entire processing workflow from screening, variant calling and exclusion of individual samples to the compilation of the investigated study cohorts is illustrated in Supplementary Fig. 1. All patients included in the present study received first-line treatment according to the respective therapeutic recommendations of the German Hodgkin lymphoma study group (GHSG) at the time but outside the scope of a clinical trial (due to temporarily limited access to clinical trials at the respective centers).

Further details on clinicopathological workup are depicted in Supplementary Material.

### Targeted hybrid capture sequencing

For targeted next-generation sequencing (tNGS), we employed the NovoPM™ 2.0 Cancer Panel (Novogene Co., Ltd., Cambridge, UK) encompassing all coding exons of 484 genes, 159 single nucleotide variants (SNV)/short insertions and deletions (indels) (Supplementary Table 1). Library preparation was carried out according to manufacturers’ instructions and sequencing was performed on the Illumina NovaSeq 6000 platform (Illumina, San Diego, California, USA) to an average depth of 8,698 (s.d. ± 2,953; median 9,094). Targeted sequencing of all 84 cHL-samples was attempted by a hybrid capture approach by Novogene (UK) Co., Ltd. Library preparation failed predefined QC metrics in one sample, 5 samples failed to produce sufficient data output in sequencing and 19 samples had to be excluded due to an intolerable fraction of sequencing quality/low-quality reads (see below). The overall sequencing approach was designed referring to the work of Schuhmacher et al*.* with an appreciation of the methodological uncertainties of analyzing cHL [15]. In complement to ueia-deep sequencing, we conducted a Pearson correlation analysis aiming to attribute the identified alterations to cHL. This involved correlating the median variant allele frequency (VAF) of each sample with the respective proportion of Hodgkin cells. To ensure precision, quantification of Hodgkin cells was performed through histopathological review of HE sections and immunohistochemically stained CD30 sections, adhering rigorously to a four-eye principle.

### Sequencing data processing, variant calling, and filtering

Raw sequencing data (paired-end fastq files) were mapped to the human genome version GRCh38 and processed using nfcore/sarek (v3.0) [16, 17]. Briefly, sequencing quality was assessed using fastqc and low-quality bases/reads were removed utilizing fastp. Next, cleaned reads were mapped to GRCh38 using bwa-mem2, and mappings were processed following GATKs best practices. Realignment of reads (marked duplicated bam files from sarek) was performed by applying ABRA2 (v3.0) [18]. Resulting realigned reads were used for multi-sample somatic variant calling of very deep next-generation sequencing data (needlestack v1.1) [19]. For further details on nucleic acid extraction, panel sequencing, variant calling please see Supplementary Methods and Supplementary Fig. 1.

### Network propagation

The effect of strongly deleterious mutations (CADD1.6 phred score > 20) on neighboring genes was assessed per sample using a network propagation approach with a regularized Laplacian kernel based on STRINGdb v11 protein–protein interaction network as implemented in the diffuStats R package (v1.20.0) [20, 21]. Mutated genes were set to 1, whereas non-mutated genes were set to 0 to model the behavior of strongly deleterious mutations. Additionally, the filtered variants were classified using AlphaMissense to assess their potential pathogenicity [22]. Network propagation was performed using a permutation-adjusted score with statistical normalization that takes into account both the magnitude of the raw scores (0 or 1) and the effect of the network topology (*Sp*—score) [23]. Estimated propagation scores (*Sp*) were used for gene set enrichment analysis against KEGG, REACTOME and/or HALLMARK gene sets (MSigDB v7.4) using gage (v2.50.0) and pathways with a Benjamini–Hochberg corrected p-value below 0.05 (q-value) were considered as significantly enriched between conditions [24].

### Assessment of positive selection

The normalized ratio of non-synonymous to synonymous mutations (*d*_*N*_*/d*_*S*_) was estimated groupwise using the R-package dndscv (v0.0.1.0) and genes showing signals of positive selection were selected if the ration was larger than 1 and the adjusted p-value was below 0.1 [25]. Differences in *dN/dS* between groups were assessed by applying Kruskal–Wallis test with Wilcoxon test as a *post-hoc* test (with Bonferroni correction to correct for multiple testing). Genes with signals of positive selection were further investigated by performing an over-representation analysis (ORA) of these genes against REACTOME gene sets (MSigDB v7.4) using a hypergeometric test. As the gene panel consists only of 484 genes, only these genes were used as background genes (see Wijesooriya et al*.* for further details [26]).

### Sequence of analyses

Primary, we assessed the mutational landscape in a subgroup of responders who received polychemotherapy and went into ongoing treatment- and relapse-free remission. Second, the mutational landscape of rHL and prHL was investigated. Third, collected genomic characteristics (TMB, enrichment analysis of non-synonymous variants, gene set enrichment analysis) were compared between the three clinical subgroups to highlight specific features with prognostic implications that provide mechanistic insights into the molecular underpinnings of the distinct clinical course. Fourth, we analyzed the longitudinal mutational dynamics of the included relapsed/refractory cases comparatively between the pretherapeutic and the post-therapeutic setting.

### Statistical analysis

All statistical analyses were performed using R (v4.3.1) and tidyverse (v2.0.0) for data handling. Filtering of genomic regions was performed using the GenomicRanges R (v1.52.0) package and data was visualized using maftools (v2.17.0), ggplot2 (v3.4.3), and ggpubr (0.6.0). For oncoplot creation, known oncogenes [27] and tumor suppressor genes [27] were selected as well as actionable genes from OncoKB (71 genes).

### Data availability

BAM files have been deposited in the European genome-phenome archive (EGA) under the accession number EGA50000000141.

## Results

### Clinicopathological characteristics

We selected 59 diagnostic samples (20 responders, 13 prHL, 26 rHL) from 44 cHL-patients with sufficient FFPE tissue available for molecular studies (median age 42 years; range 18 – 80 years) all of which were included in the final analysis after successful library preparation for tNGS. 39 samples were from initial diagnosis and 20 samples were from disease relapse/progression after polychemotherapy and in 19 cases consolidating radiation. We acknowledge limited clinical follow-up in two prHL and one rHL (3/56; 5.4%). Most patients were male (27/44; 61%) and many presented with advanced-stage disease (18/41 had Ann Arbor III/IV; 44%). A proportionate number of patients had extranodal manifestation at diagnosis regardless of the clinical subgroup (20/44; 49%). In total, 11/41 (27%) patients presented with a mediastinal bulk. Manifestations in at least three nodal sites were detected in 22/41 (54%) cHL patients. Risk stratification of cHL patients according to the Hasenclever-IPS categorized most patients into the low-risk group (0–1: 15/41; 37%) or the intermediate-low risk group (2–3: 14/41; 34%), respectively. Among responders, there were significantly (*p* = 0.021; X2 = 5.304) fewer cases categorized as high-intermediate/high according to the Hasenclever IPS (Supplementary Table 2). The Supplementary Figs. 2 – 4 present three illustrative clinical courses from the cohort, highlighting the variability in disease progression. These selected cases underscore the heterogeneity of clinical trajectories and the challenges posed by tumor biology, particularly in instances of recurrence or refractory disease.

The most common histologic cHL subtype was nodular sclerosis (23/44; 52%) followed by cHL of mixed cellularity (15/44; 34%). Upon EBER in situ hybridization, 18/42 (43%) were EBV-positive (nodular sclerosis 4/23; mixed cellularity 11/15). One patient refused systemic polychemotherapy therapy (2%) and 19/41 (46%) received consolidative radiation.

Baseline clinicopathological characteristics for each clinical subgroup (responders versus rHL versus prHL) are summarized in Table 1. The clinical course of each case of the study cohort is illustrated in Supplementary Fig. 5.

**Table 1:** Baseline clinicopathological characteristics of the study cohort

Characteristics		Responder cHL (*n* = 20) *	cHL with relapse (*n* = 16) **	Primary refractory cHL (*n* = 8) ***
Sex	Male	12 (60%)	10 (63%)	5 (63%)
	Female	8 (40%)	6 (37%)	3 (37%)
Age	Median (range)	40 (20 – 80)	50 (18 – 68)	53 (20 – 75)
BMI	< 25 kg/m^2^	15 (75%)	9 (56%)	8 (100%)
	25 – 29.9 kg/m^2^	4 (20%)	7 (44%)	-
	30 – 34.9 kg/m^2^	1 (5%)	-	-
Ann Arbor stage	I	-	-	-
	II	13 (65%)	6 (40%)	4 (67%)
	III	3 (15%)	4 (27%)	1 (17%)
	IV	4 (20%)	5 (33%)	1 (17%)
B-symptoms	Yes	8 (40%)	8 (53%)	3 (50%)
	No	12 (60%)	7 (47%)	3 (50%)
LDH	Elevated	7 (35%)	3 (20%)	4 (67%)
	Normal	13 (65%)	12 (80%)	2 (33%)
ECOG-PS	0 – 1	16 (80%)	12 (80%)	4 (67%)
	> 1	4 (20%)	3 (20%)	2 (33%)
Extranodal sites	0	11 (55%)	8 (53%)	2 (33%)
	1 – 2	8 (40%)	7 (47%)	3 (50%)
	> 2	1 (5%)	-	1 (17%)
GSHG risk factors	Mediastinal bulk	4 (20%)	5 (33%)	2 (33%)
	Extranodal disease	9 (45%)	7 (47%)	4 (67%)
	≥ 3 involved sites	10 (50%)	9 (60%)	3 (50%)
IPS Hasenclever	0—1	10 (50%)	4 (27%)	1 (17%)
	2 – 3	8 (40%)	5 (33%)	1 (17%)
	4	1 (5%)	4 (27%)	3 (50%)
	5—7	1 (5%)	2 (13%)	1 (17%)
CCI	0—2	13 (65%)	10 (66%)	1 (17%)
	> 2	7 (35%)	5 (33%)	5 (83%)
Histological subtype	Nodular sclerosis	11 (55%)	11 (73%)	2 (33%)
	Mixed cellularity	9 (39%)	2 (13%)	4 (67%)
	Lymphocyte-depleted	-	-	-
	Lymphocyte-enriched	-	1 (6%)	1 (17%)
	Not further specified	-	2 (13%)	1 (17%)
EBV-status	Positive	8 (40%)	6 (44%)	4 (50%)
	Negative	12 (60%)	9 (56%)	4 (50%)
1st line treatment	ABVD or COPP	8 (40%)	3 (20%)	2 (33%)
	BEACOPP esc. + ABVD	4 (20%)	1 (6%)	-
	BEAACOPP esc	5 (25%)	8 (53%)	2 (33%)
	other	2 (10%)	3 (20%)	2 (33%)
	Refused cytoreduction	1 (5%)	-	-
Consolidation radiation	Yes	9 (45%)	7 (47%)	3 (50%)
	No	11 (55%)	8 (53%)	3 (50%)
1st line best response	CR	14 (75%)	4 (27%)	-
	PR	5 (25%)	10 (66%)	-
	SD	-	1 (6%)	5 (71%)
	PD	-	-	2 (29%)

*LDH* lactate dehydrogenase, *ABVD* doxorubicin, bleomycin, vinblastine, dacarbazine, *COPP* cyclophosphamide, vincristine, procarbazine, prednisone, *BEACOPP* bleomycin, etoposide, doxorubicin, cyclophosphamide, vincristine, procarbazine, prednisone, *CCI* Charlson comorbidity index, *ECOG* Eastern cooperative oncology group

^*^clinical dataset was fully available in 20 patients

^**^clinical dataset was fully available in 15/16 patients

^***^clinical dataset was fully available in 6/8 patients

### The mutational spectrum of classic Hodgkin lymphoma irrespective of the clinical course

**Fig. 1:**
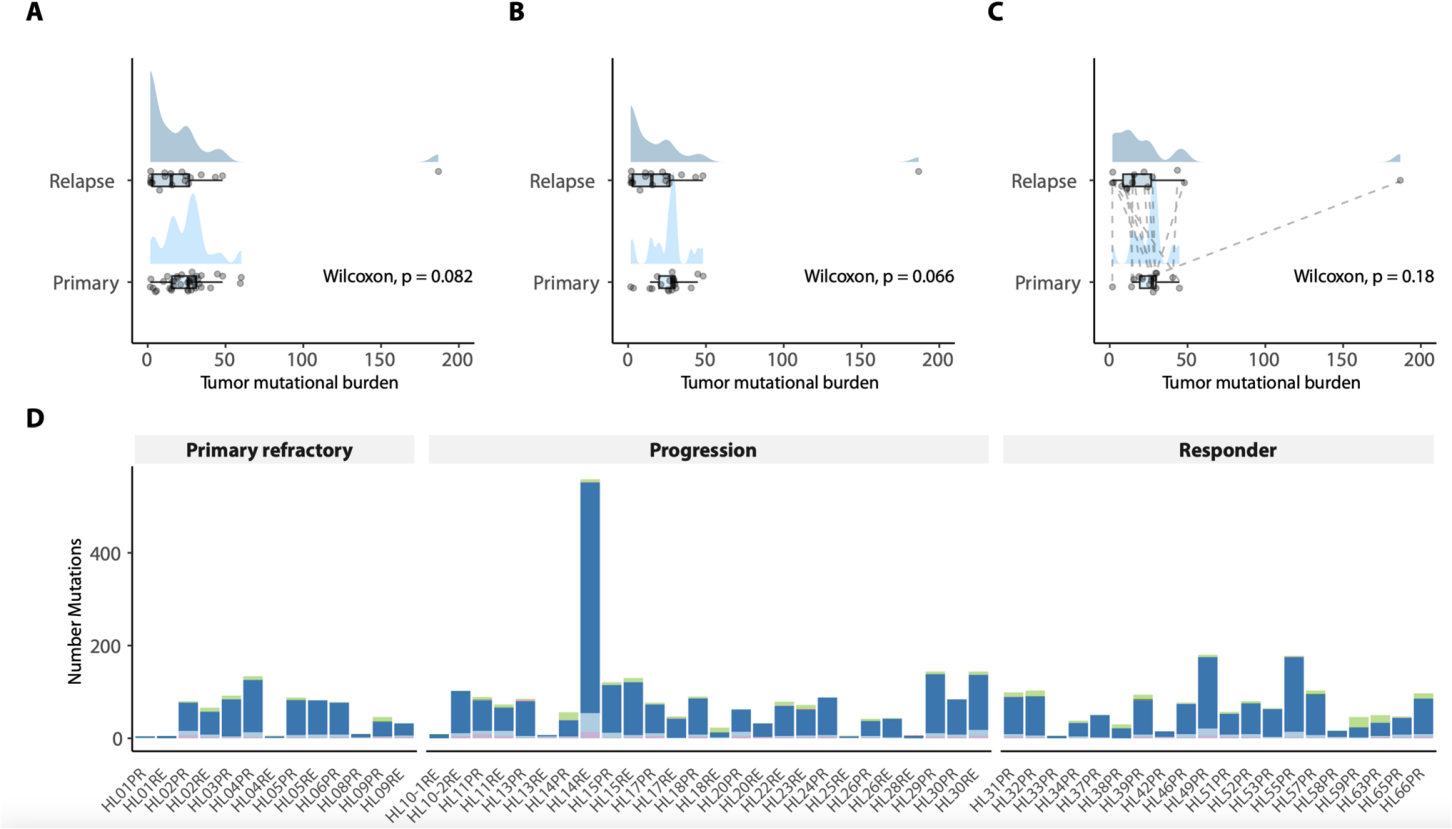
Comparative TMB analysis as well as number and type of mutations. **A** shows the TMB comparison between all samples from the initial diagnosis and all samples from the relapse/progression setting. **B** Here, only samples from progression and primary refractory were considered. **C** In this part of the figure only matched sample pairs from the initial diagnosis and relapse/progression setting were considered. Each panel (**A**-**C**) shows the density of the data as well as each data point in addition to median and interquartile range (boxplot). **D** delineates the number and subtypes of genetic alterations for each included sample ordered by the clinical course of cHL

As described in the previous section, the mutational landscape was analyzed on a total of 59 samples from 44 cases. All cHL cases carried mutations of oncogenic relevance according to our bioinformatic annotations. The median tumor mutational burden (TMB) was 50.50 mutations/MB (mean: 61.19; standard deviation ± 74.8). Comparative TMB analysis revealed no significant differences in TMB between primary and relapse samples (Wilcoxon test *p* = 0.082) or with progression type (*p* = 0.18) (Fig. 1 A–C). In total, we identified 4,432 non-silent mutations in 450 genes as well as 304 frame-shift indels and 13 in-frame indels (total 317 indels; 7.2%). In detail, the spectrum of mutations covered 3,676 missense mutations (82.9%), 289 nonsense mutations (6.5%), 150 splice-site mutations (3.4%) (Fig. 1D). Utilizing an assessment methodology following the "four-eye principle," we quantified the Hodgkin cell content in each sample. The subsequent correlation analysis unveiled a notable association between the Hodgkin cell count in the specimens and the median VAF of detected alterations within each sample (Pearson correlation coefficient r = 0.6123, *p* < 0.0001; Supplementary Fig. 6). This substantiates the attribution of the identified alterations to cHL.

### Comparative analysis of mutational landscape in primary refractory, relapsed cHL and responders

**Fig. 2:**
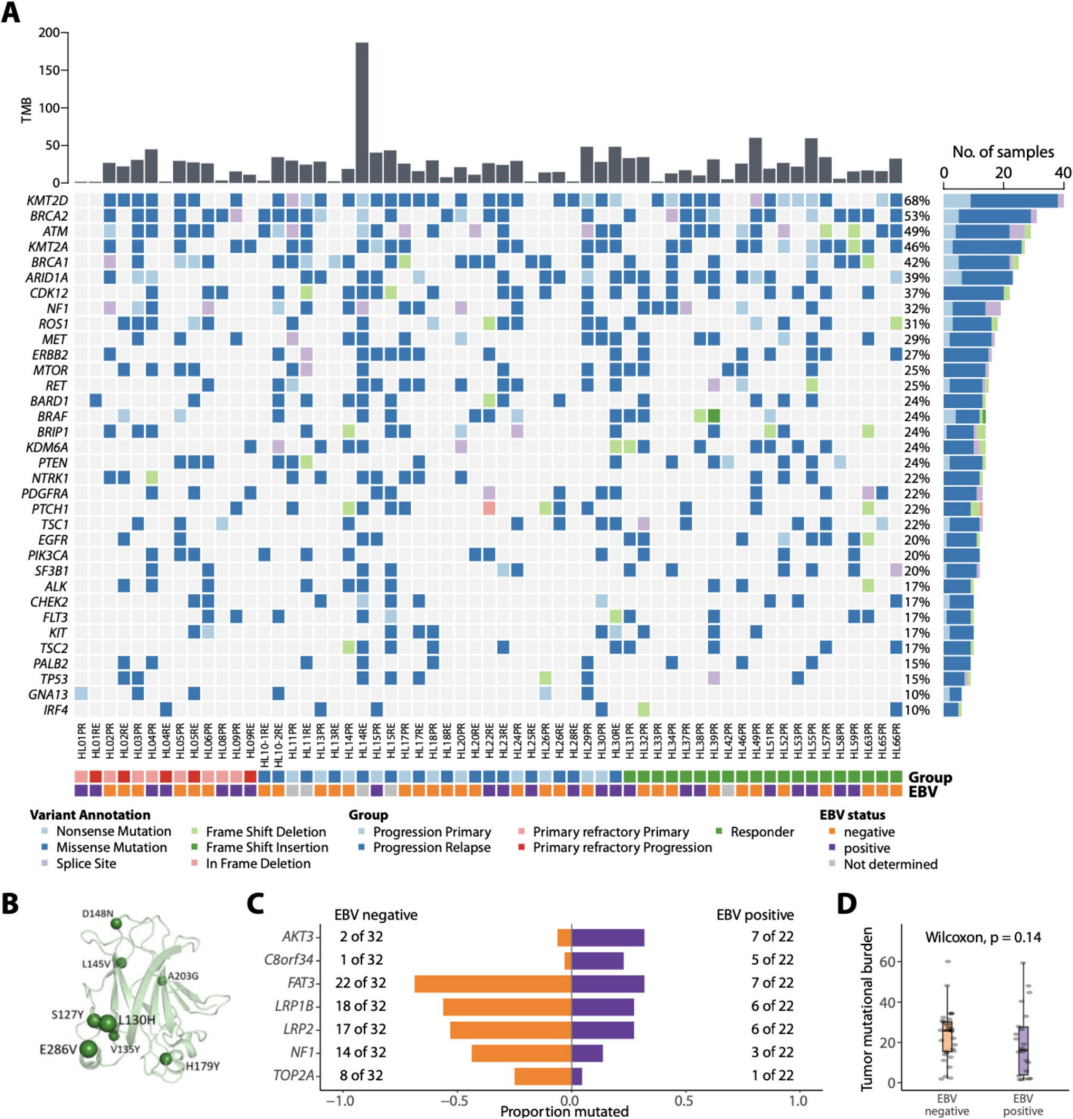
Mutational landscape in HL. **A** The oncoplot illustrates the mutational landscape of the most severe mutations in our cHL cohort including primary refractory cases (prHL: HL01—HL09), cases experiencing relapse after initially responding towards standard polychemotherapy (rHL: HL10—HL30), and responders (responder: HL31—HL66). The oncoplot also includes the EBV status of each case. **B** PyMol plot delineates the 3-dimensional structure of TP53, and a relevant proportion of alterations found in our sequencing approach. All mutations demonstrated in this plot affect die DNA binding site. Mutations that significantly differed between EBV-positive and EBV-negative cases are shown in (**C**) and (**D**) demonstrating a trend towards a higher TMB in EBV-negative samples compared to EBV-negative samples

**Fig. 3:**
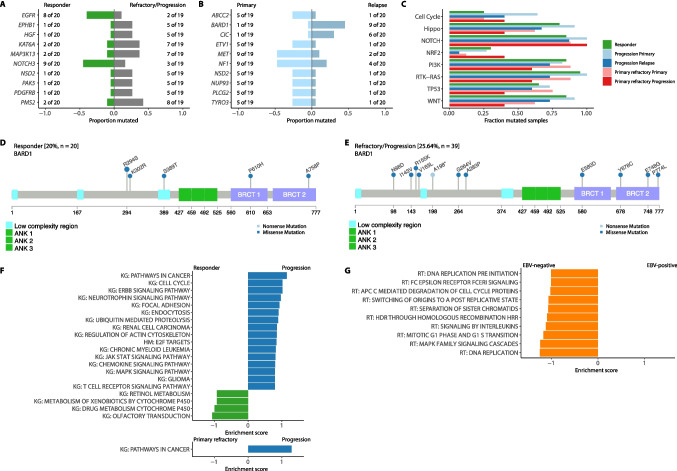
Clonal evolution and implicated pathways in relapsed/refractory HL. **A** The distinct mutational profile between responders and cases experiencing relapses/progression (prHL + rHL). **B** illustrates the longitudinal trajectory of cases in which relapses/primary-refractory disease occurred. **C** The bar plot shows the fractions of mutated pathways in a comparative manner within the spectrum of clinical courses regarded here. **D** Lollipop plot visualizes the position of genetic alterations within the gene *BARD1* in responders and (**E**) primary-refractory/relapsed cases to infer their functional relevance. **F** Gene set enrichment of network propagation analysis comparing baseline samples from responders (green; negative enrichment scores) and primary-refractory/relapsed cases (blue; positive enrichment scores) against KEGG (KG) and HALLMARK (HM) pathways as well as (**G**) comparing EBV-positive (no significantly enriched pathways) and EBV-negative cases (orange; negative enrichment scores) against REACTOME (RT) gene sets

The most commonly mutated genes ordered by clinical course and EBV-status are illustrated in Fig. 2A (Supplementary Fig. 7). The most frequently mutated gene was *KMT2D* (68%). This mutation occurred irrespective of the clinical course of cHL. The list of genomic alterations includes several genes previously implicated in cHL-pathogenesis such as *ARID1A* (39%), *TP53* (15%), *JAK2* (10%), *XPO1* (5%) and others (*GNA13, TNFAIP3, STAT3, NFKBIA, CARD11, TET2, BTK, SOCS1,* and *KDM5A)*. The majority of *TP53*-mutations detected here, affect the DNA binding domain (exon 4, aa109 – aa288), indicating the pathogenetic significance and deleterious character of the aberrations (Fig. 2B). In several ontogenically relevant genes such as *PIK3CA,* we exclusively detected mutations affecting functionally relevant amino acid sequences (Supplementary Fig. 8). Mutations in *AKT3* and *C8orf34* were significantly enriched in EBV-positive cases whereas mutations in *FAT3, LRB1B, LRP2, NF1* and *TOP2A* were predominantly identified in EBV-negative cHL, hinting at a partially distinct molecular pathogenesis (Fig. 2C). In EBV-negative cases, *FAT3* mutations were detected most frequently (69%; 22/32 samples). This is in line with results from an ultra-deep sequencing approach in cHL [28]. Moreover, we observed no statistically significant difference in TMB between EBV-positive and negative cases in our limited cohort (Fig. 2D). Upon comparison between responders and rHL/prHL samples at initial diagnosis, we observed a significant enrichment of mutations affecting *PMS2, PDGFRB, PAK5, NSD2, KAT6A, EPHB1, HGF* and *MAP3K13* in the latter group (Fig. 3A). For a relevant subset of such alterations, the change in the tertiary structure of the corresponding molecules was analyzed in order to illustrate the biological relevance of these mutations (Supplementary Fig. 9). In addition, each specific alteration underwent individual annotation for its functional relevance in accordance with the board certified UCCSH molecular tumor board pipeline as previously reported by our group (Supplementary Table 3) [29, 30]. Concurrently, mutations in *EGFR* and *NOTCH3* were enriched in responders (Fig. 3A). A comprehensive description of all variants described by panel based NGS is provided in the Supplementary Table 4*.*

### Longitudinal dynamics and clonal evolution in relapsed/refractory cHL

Subsequently, we analyzed the mutational trajectories between primary diagnostic samples and subsequent biopsies from rHL/prHL. Hereby, we observed a significant increase in *BARD1* and *CIC* mutations whereas *ABCC2, ETV1, NF1, NSD2, NUP93, PLCG2, MET* and *TYRO3* mutations were significantly depleted upon disease progression following polychemotherapy (Fig. 3B). We found a nearly similar distribution regarding the localization of mutations in *BARD1* between responders and rHL/prHL cases across the functional structure of the gene (Figs. 3D and E). However, *BARD1* mutations occurred more frequently in rHL/prHL cases (20% versus 26%). Interestingly, we found *BARD1* mutations in 20% of responders but only in 5% of rHL/prHL cases at initial diagnosis. In rHL/prHL cases, *BARD1* mutations do not appear until progression (5% versus 45%; Fig. 3B). Longitudinal mutational profiling further revealed a significant shift in mutationally impaired pathways with an apparent selection process in favor of NOTCH-pathway mutations as putative drivers of rHL/pr/HL in contrast to an overall decrease in mutations affecting most other pathways (Fig. 3C). A network propagation approach underscored the distinct mutational profile between responders and baseline samples from rHL/prHL cases. We found that mutations in cases developing unfavorable clinical courses were significantly enriched in cell cycle regulators, focal adhesions, MAPK signaling, and IL6/JAK/STAT3 signaling as well as T-cell receptor signaling, whereas in responders there was an accumulation of mutations affecting genes of the hepatic cytochrome P450-complex. Interestingly, mutations in typical cancer pathways were more frequently assigned to rHL cases and relatively rare in cases experiencing prHL (Fig. 3F). Supplementary Table 6 depicts at which points in the clinical course the detected alterations occurred. Comparing EBV-positive with EBV-negative cHL upon our network propagation approach revealed a significant enrichment of mutations involved in DNA replication, cell cycle regulation, MAPK signaling, DNA mismatch repair via homologous recombination deficiency (HRD), and interleukin-mediated inflammation in EBV-negative cHL (Fig. 3G). This confirms the distinct pathogenesis shaped by an underlying EBV-infection. Additionally, we specifically emphasize the role of APOBEC gene signature that has previously been reported by others (Supplementary Fig. 10) [11, 31].

### Positive selection analysis

**Fig. 4:**
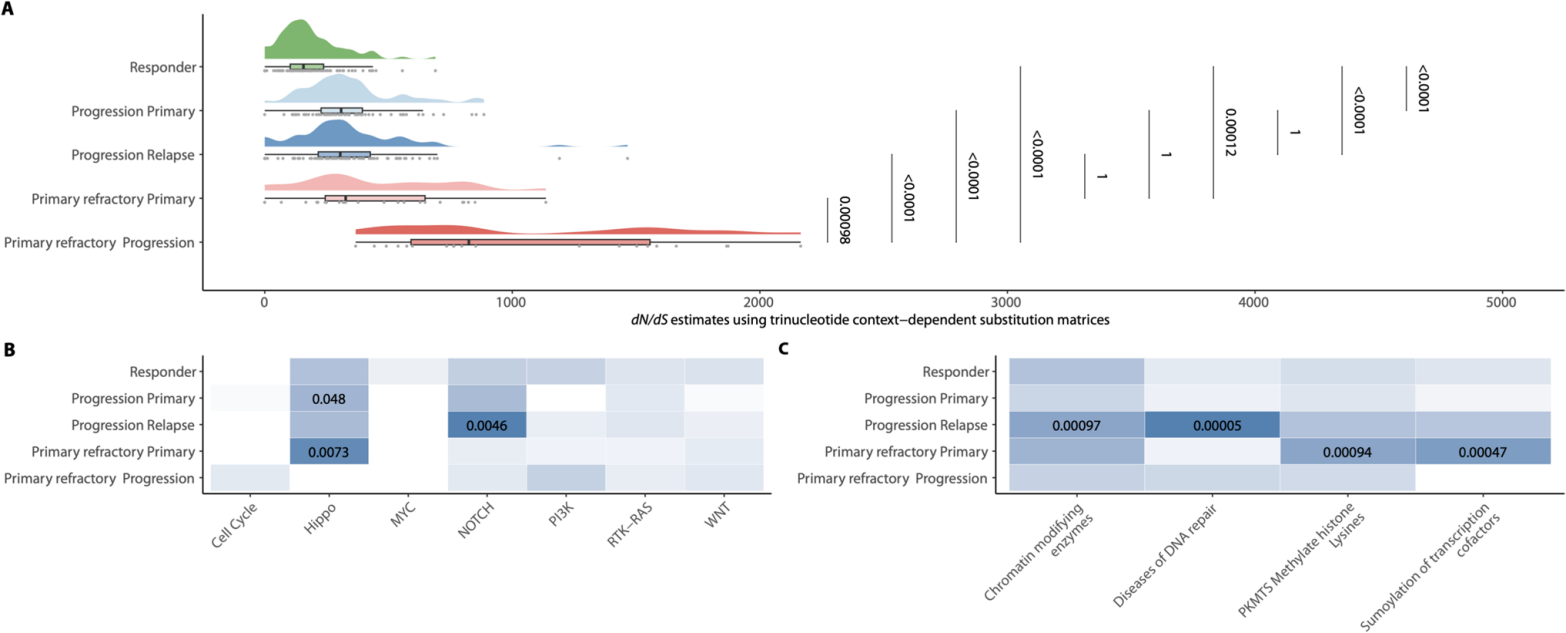
Positive selection analysis in cHL. **A** Distribution of *dN/dS* estimates for genes showing a significantly increased ration (q < 0.1; Wilcoxon test results from pairwise comparisons are added); panel shows the density of the data as well as each data point in addition to median and interquartile range (boxplot). The heatmaps depict *p*-values of overrepresentation analysis against oncogenic pathways (*p* < 0.05) (**B**) and REACTOME gene sets (*p* < 0.001) (**C**); for significantly overrepresented terms *p*-values values are shown

Further, we investigated the mutational trajectories for positive selection pressure under chemotherapy regarding the distribution of synonymous versus non-synonymous variants. Hereby, we observed a significant enrichment of non-synonymous variants in both prHL patients and rHL patients compared to responders in front-line therapy setting. In addition, we observed a further significant enrichment of non-synonymous variants upon disease progression in prHL, which was not observed in late relapses (Fig. 4A). Supplementary Table 5 provides the number of significantly mutated genes showing positive selection and mean values of dn/ds ratios for each clinical setting that was evaluated here. Moreover, we performed an overrepresentation analysis of genes with a significantly increased dN/dS ratio (q < 0.1). In particular, mutations in the hippo pathway led to an enrichment of non-synonymous variants in samples from initial diagnosis in which recurrence/progression occurs later in the course of disease (rHL). Interestingly, these alterations appear to undergo negative selection, as they tend to disappear at the time of relapse or progression.

In a recently published deep sequencing approach, the pathogenetic role of the hippo pathway has already been elaborated in cHL [28]. Our dataset supports this role. In contrast, a positive selection process can also be detected in genes inherent to NOTCH signaling. In prHL, this selection cannot be detected neither at initial diagnosis nor in progression (Fig. 4B). Testing against REACTOME revealed a positive selection of non-synonymous variants affecting genes of DNA repair damage and chromatin-modifying enzymes in the relapse setting as well as the regulation of methylation and sumoylation of transcription co-factors in prHL (Fig. 4C).

### Survival analysis

In an exploratory integrated analysis of clinical and molecular data on our preselected cohort, the prognostic impact of genetic alterations on progression-free survival (PFS) and overall survival (OS) of cHL patients was investigated. Here, we focused on mutations present with a frequency of at least 20% in the study cohort (25 genes) and mutations that play a critical role in the clonal evolution of the disease (*BARD1, NF1, MET*). Bonferroni’s correction for multiple testing was omitted. Here, alterations in *BRCA1* were found to be significantly predictive for both unfavorable PFS (*p* = 0.028, HR = 3.19) and OS (*p* = 0.032, HR = 3.19) at initial diagnosis. In addition, mutations in *BRIP1* were identified to predict unfavorable OS (*p* < 0.001, HR = 15.9) but not PFS. The detection of *ERBB2* (*p* = 0.021, HR = 0.235) and *BRAF* (*p* = 0.049, HR = 0.305) mutations in samples from relapse/progression setting were found to predict favorable PFS (Supplementary Fig. 11). Using a Cox proportional hazard model, we investigated the independent prognostic value of molecular markers that were prognostically relevant for PFS and/or OS upon Kaplan Meier analysis (Supplementary Fig. 12). These trends could be confirmed for *BRCA1* for PFS (*p* = 0.029, HR = 3.54), *BRAF* (PFS: *p* = 0.036, HR = 0.14) and for *BRIP1* in regard to OS (*p* = 0.013, HR = 27.79). For *BRCA1* OS (*p* = 0.180, HR = 4.79) and *ERBB2* PFS (*p* = 0.063, HR = 0.19), the trends could not be confirmed. The multivariate analysis included clinical parameters that are considered established independent risk factors for predicting the survival of cHL patients.

## Discussion

This study investigates the mutational landscape of prHL and rHL compared to responders using extensive hybrid capture NGS diagnostics. For this purpose, samples from untreated cHL cases as well as post-therapeutic samples from rHL or prHL patients were analysed. A total of 15 paired tissue samples from the time of initial diagnosis and from progression were available. To date and to the best of our knowledge, this is the most comprehensive approach to analyze the genomic characteristics of this difficult-to-treat subgroup of cHL patients. In this work, we made three major observations.

First, our analyses reveal that the mutational landscape of rHL/prHL differs from that of responders. Consistent with the results of previous genetic characterizations of cHL, we found nine events of *SOCS1* alterations among eight cases. In addition, we detected further driver mutations in the genes *TP53, GNA13, KMT2D, TNFAIP3, JAK2, STAT3, XPO1, NFKBIA, CARD11, TET2, BTK, ARID1A* and *KDM5A* in our data set at initial diagnosis, which have been described as typical in cHL. Some frequent alterations such as *STAT6* or *IGLL5* are not covered by our sequencing panel and are therefore beyond the scope of this work [11, 31–33]. Additional eight mutations that were significantly enriched in rHL/prHL affect genes that were previously associated with the progression of other lymphoma subtypes (e.g., *PDGFRB, KAT6A, HGF, EPHB1, NSD2, PMS2*) [30, 34–36]. A previous study reported that *EPHB1*, a receptor tyrosine kinase, is overexpressed through the activation by autocrine loops in cHL [37]. Interestingly, *PMS2* mutations were previously associated with the occurrence of secondary malignancies such as colorectal carcinoma in cHL survivors after systemic polychemotherapy [38, 39]. Additionally, our analyses reveal several therapeutic vulnerabilities (*PDGFRB, NTRK1/2/3, RET, BRCA1/2, PIK3CA, BRAF, mTOR, ERBB2, EGFR, FLT3, KIT, CDKN2A, IDH1/2, JAK2, EZH2, FGFR1/2/3*) for this difficult-to-treat patient population that may critically expand the future spectrum of therapeutic options. Overall, the distinct genomic signature of rHL/prHL suggests an independent tumor biology.

Second, the sequential genomic workup of paired tissue samples from initial diagnosis and progression underscores the impact that systemic polychemotherapy has on the constellation of genetic alterations. Thus, *BARD1* mutations can be detected significantly more frequently after polychemotherapy and to a lesser extent in untreated cHL. The present data suggest that these mutations play a role in the development of resistance towards chemotherapy and subsequently lead to rapid clonal selection and expansion of the mutant cell fraction. Functionally, mutations in *BARD1* belong to the group of mutated genes such as *BRCA1/2, CHEK2, CDH1, PALB2, RAD51C, RAD51D* and *TP53* leading to HRD [40, 41]. Such genes play an essential role in DNA damage response (DDR) [42]. Previously, *BARD1* mutations were already assigned to the HRD signature for other entities [43–45]. Moreover, the supporting role of *BARD1* for cellular viability has already been demonstrated in preclinical approaches [46–48]. Most importantly, the almost exclusively post-therapeutic occurrence suggests that HRD plays a supporting role in the formation of resistance mechanisms. This assumption is supported by the concurrently frequent detectable alterations which we found in *BRCA2 53%, BRCA1 42%, CHEK2 15%, PALB2 15%, TP53 15%, RAD51D 7% and RAD51C 5%.*

In addition to the expansion of *BARD1* mutations, we observed that some mutations are compressed in the progressive disease setting (*ABCC2, ETV1, NF1, NSD2, NUP93, PLCG2, MET*, *TYRO3*).

The loss of such mutations in the rHL/prHL appears congruent with the third major finding of this work, the clonal selection of NOTCH-associated mutations and the concomitant repression of other signalling pathways that were previously suggested to be responsible for lymphomagenesis of cHL (IL6/JAK/STAT, NF-KB, PI3K/AKT/mTOR). It is well known that the NOTCH pathway is involved in the pathophysiology of cHL [49]. Of particular note is the hierarchical role that this pathway plays during the selection process within the progress of the disease which we detected in the present study. The pathway is involved in natural B cell development [50]. In cHL, overactivation of NOTCH signalling has been reported [51]. Particularly in EBV-positive cHL cases, NOTCH overactivation can synergistically trigger NF-KB signaling [51].

Recently, the feasibility of liquid biopsy approaches via the analysis of cfDNA in cHL was impressively demonstrated. These approaches are able to successfully reproduce the mutational landscape of cHL [13]. Searching for *BARD1* mutations under polychemotherapy in cHL patients by liquid biopsy may serve as a promising parameter for early treatment failure detection in a relevant subset of cHL patients. However, the identification of *BARD1* mutations does not serve as a surrogate parameter for minimal residual disease (MRD) diagnostics. Although our data indicate the occurrence of *BARD1* mutations in the disease progression of cHL-patients with adverse outcomes, there’s an absence of evidence linking the disappearance of such mutations to therapeutic responses. Simultaneous selection of alterations affecting NOTCH signalling may contribute to the detection of primary-refractory disease (prHL) progression. Thus, the analysis of cfDNA may play a major role in future treatment guidance.

Beside the possible applications of liquid biopsy, the era of precision oncology is characterized by molecularly stratified targeted cancer therapies [52]. The beforementioned diversity of targeted treatment options again underscores that the inclusion of relapsed/refractory aggressive lymphomas in molecular tumour boards may pose a promising option for such patients [29, 30].

Limitations of the present study are those inherent to its retrospective design and limited sample size. Therefore, our results require prospective validation in a larger cohort, treated within the scope of a PET-guided prospective trial. Sequencing of sorted Hodgkin/Reed-Sternberg cells would have been desirable for this study. In order to assign mutagenic events to cHL, we selected an ultra-deep sequencing approach applying a comprehensive, commercially available gene panel. Employing a more comprehensive sequencing method such as whole exome or genome sequencing could have offered deeper insights into the diverse genetic profiles characterizing various clinical trajectories of cHL. However, the scope of our current study precluded the utilization of these approaches. Our primary concern was maintaining high sequencing quality, particularly given the occasional low content of Hodgkin cells in the samples. Therefore, we chose panel sequencing with a high coverage to ensure robustness. Moreover, the selected panel encompassed a wide array of potential driver genes previously implicated in cHL and other hematologic neoplasms.

Pairing of our panel-sequencing results with RNA-sequencing data, the correlation with deep-sequencing of cfDNA and further integration of our results into the context of the molecular clusters considering comprehensive SNV analysis, preferably in an extended, clinically annotated cohort, which was beyond the scope of the present study, would further deepen our molecular understanding of rHL/prHL especially regarding EBV-positive cases. The treatment landscape within the analyzed cohort exhibits considerable heterogeneity. While a subset of cases aligns with the era incorporating novel immunotherapeutic agents (e.g., nivolumab) or targeted therapies (e.g., brentuximab vedotin), markedly impacting cHL therapy in recent years, a significant portion of patients underwent treatment preceding the introduction of these agents. This prompts speculation regarding the potential reduction in the proportion of patients experiencing relapsed/refractory disease in subsequent periods. Definitively answering this query remains elusive. Nonetheless, conventional chemotherapy protocols like ABVD and/or BEACOPP-based strategies continue to hold relevance as fundamental components in the first-line treatment approach for cHL.

In summary, we delineate the mutational landscape in addition to longitudinal dynamics of mutational profiles of rHL/prHL and comprehensively explore its similarities and distinctions, compared with responders in cHL, including several exclusive oncogenic drivers. Moreover, we provide an overview of targetable mutational vulnerabilities and drivers of refractory disease as a roadmap for future efforts toward individualized treatment strategies in cHL. Our analyses show that *BARD1* mutations occur with the progression of cHL after polychemotherapy. Indirectly, this suggests that acquired *BARD1* mutations in rHL/prHL are critically involved in the development of resistance mechanisms. Furthermore, in prHL there is positive selection towards mutations affecting NOTCH-signaling with concurrent depletion of mutations in other pathways characteristic for cHL.

## Supplementary Information

Below is the link to the electronic supplementary material.

Supplementary file1 (DOCX 25776 KB)

Supplementary file2 (XLSX 15 KB)

Supplementary file3 (XLSX 25 KB)

Supplementary file4 (XLSX 903 KB)

Supplementary file5 (XLSX 10 KB)

## Data Availability

BAM files have been deposited in the European genome-phenome archive (EGA) under the accession number EGA50000000141.
