# Supplementary material for: The mutational landscape and its longitudinal dynamics in relapsed and refractory classic Hodgkin lymphoma
Source: Ann Hematol. 2025 Feb 24;104(3):1721–33. doi: 10.1007/s00277-025-06274-5 (PMC12031843; doi:10.1007/s00277-025-06274-5)
Supplement: Supplementary file 1 — Supplementary file1 (DOCX 25776 KB) [file 277_2025_6274_MOESM1_ESM.docx]

**Title: The mutational landscape and its longitudinal dynamics in relapsed and refractory classic Hodgkin lymphoma**

**Authors:** Hanno Witte^1,2,3^*, Axel Künstner^1,4,5*^, Thomas Hahn^1,3,6^*, Veronica Bernard^6^, Stephanie Stölting^6^, Kathrin Kusch^6^, Kumar Nagarathinam^7^, Cyrus Khandanpour^1,3^, Nikolas von Bubnoff^1,3^, Arthur Bauer^2^, Michael Grunert^8^, Svenja Hartung^9^, Annette Arndt^10^, Konrad Steinestel^10^, Hartmut Merz^6^, Hauke Busch^1,4,5**^, Alfred C. Feller^6**^, Niklas Gebauer^1,3**^

**Affiliations:**

^1^ University Cancer Center Schleswig-Holstein, University Hospital of Schleswig-Holstein, Campus Lübeck, 23538 Lübeck, Germany

^2^ Department of Hematology and Oncology, Bundeswehrkrankenhaus Ulm, Oberer Eselsberg 40, 89081 Ulm

^3^ Department of Hematology and Oncology, University Hospital of Schleswig-Holstein, Campus Lübeck, Ratzeburger Allee 160, 23538 Lübeck, Germany

^4^ Medical Systems Biology Group, University of Lübeck, Ratzeburger Allee 160, 23538 Lübeck, Germany

^5^ Institute for Cardiogenetics, University of Lübeck, Ratzeburger Allee 160, 23538 Lübeck, Germany

^6^ Hämatopathologie Lübeck, Reference Centre for Lymph Node Pathology and Hematopathology, Maria-Goeppert-Straße 9a, 23562 Lübeck, Germany.

^7^ Institute of Biochemistry, University of Lübeck, Ratzeburger Allee 160, 23538 Lübeck, Germany

^8^ Department of Nuclear Medicine, Bundeswehrkrankenhaus Ulm, Oberer Eselsberg 40, 89081 Ulm

^9^ Institute of Pathology, University Ulm, Albert-Einstein-Allee 23, 89081 Ulm

^10^Institute of Pathology and Molecularpathology, Bundeswehrkrankenhaus Ulm, Oberer Eselsberg 40, 89081 Ulm

**Supplementary Material**

**Supplementary Methods**

*Sample acquisition and clinicopathological characteristics*

For each case, diagnosis was confirmed by hematopathologic reference in accordance with the 4th edition of the WHO classification of tumors of the hematopoietic and lymphoid tissues^1^. From 84 samples meeting diagnostic and clinical criteria for cHL with available biopsy specimen, 59 were selected for subsequent genomic analysis, based on tumor DNA quality and library preparation success. Clinical information was collected from the original files, and patients’ performance status (Eastern Cooperative Oncology Group [ECOG]), stage, treatment modalities, therapeutic response, pattern of relapse, baseline serum levels of lactate dehydrogenase (LDH), international prognostic score (Hasenclever-IPS), GHSG risk factors and information on survival were anonymously coded alongside hematopathological assessments. Extent of disease was routinely evaluated according to the Cotswold modifications of the Ann Arbor classification^2^. EBV-status was assessed based on chromogenic *in situ* hybridization for EBER.

*Extraction of nucleic acids*

Genomic DNA was extracted from two to four FFPE tissue sections of 5-µm thickness using Maxwell ® RSC DNA FFPE kit (Promega, Wiesloch, Germany) or the QiaAmp mini kit 250 (Qiagen, Hilden, Germany), according to the manufacturers’ instructions. Quality assessment and quantification was performed on an Agilent 2100 Bioanalyzer system (Agilent Technologies).

*Sequencing data processing, variant calling and filtering*

Raw sequencing data (paired-end fastq files) were mapped to the human genome version GRCh38 and processed using nfcore/sarek (v3.0)^3,4^. Briefly, sequencing quality was assessed using fastqc and low-quality bases/reads were removed utilizing fastp. Next, cleaned reads were mapped to GRCh38 using bwa-mem2 and mappings were processed following GATKs best practices. Realignment of reads (marked duplicated bam files from sarek) was performed by applying ABRA2 (v3.0)^5^. Resulting realigned reads were used for multi-sample somatic variant calling of very deep next-generation sequencing data (needlestack v1.1)^6^. In detail, for each position and for each potential variant, a sequencing error model was fitted, and variants were detected if they were outliers from the error model. Identified variants were left-aligned (GATK v4.2.3.0)^7^ and for each sample, a vcf file was created (bcftools v1.11) and variants were annotated using Variant Effect Predictor (VEP v103, GRCh38; adding CADD v1.6, dbNSFP v4.1a, and gnomAD r3.0 as additional annotations)^8,9^ and annotations were converted into *MAF* format using vcf2maf (v1.6.21)^10^; coverage was extracted directly from the INFO field in the vcf files. Potential FFPE artifacts in the variant data was identified using two approaches. First, strand orientation biases (mutations just found on one strand, F1R2 or F2R1) were detected by SOBDetector (v1.0.4) and potential artifacts were removed^11^. Next, a classifier for detection of the origin of mutation in formalin-fixation paraffin-embedding (FFPE) samples was applied (R-package excerno v0.1.0)^12^. Variants annotated with an FFPE-like signature were removed, leaving 771,768 variants across 84 samples for further processing.

Potential germline variants in the data were removed using GATKs 1000g Panel of Normals (35,600 variants removed; 4.6% of all variants). Next, samples were checked for quality issues and one sample was removed due to an unusually high number of mutations (sample HLPR54). Variants outside known coding regions (located in e.g., intron, UTRs) were removed and variants with population allele frequency > 0.001 in GNOMAD or POPFREQ MAX database were discarded, as well as variants outside regions defined in the sequencing panel. To keep only clinically relevant variants, variants with a VAF lower than 0.001 and larger than 0.2, with a CADD score below 10, annotated as benign (CLINVAR) and without annotation in the existing variation column where removed. Finally, samples with less than 5 non-silent mutations were excluded, leaving 59 samples with 4,715 variants for downstream analyses.

*Statistical analysis*

Progression-free survival and overall survival (PFS, OS) were calculated from the date of diagnosis. Events for PFS were defined as follows: disease progression, relapse, or death from any cause, whichever occurred first. For OS, the event was defined as death from any cause. Patients who were alive without an event at the time of the last follow-up were censored at that point. Survival (PFS and OS) according to potential prognostic factors was estimated by means of the Kaplan–Meier method and univariate log-rank test. Survival analysis was carried out employing the R packages SURVIVAL (3.2-7) and SURVMINER (v0.4.8). A Pearson correlation analysis was performed to investigate the association between the median VAF of a sample and the previously counted Hodgkin cell content.

**Supplementary Tables**

**Supplementary Table 1.** List of genes included in our panel sequencing approach - see separate .xlsx file.

**Supplementary Table 2.** Chi square testing for statistical distinctions between Hasenclever IPS subgroups in our study cohort.

| **Characteristics** | **Responder**  **(n = 20) *** | **rHL/prHL**  **(n = 24) **** | **Chi-square test**  **(p-value)** | **X2 value** |
| --- | --- | --- | --- | --- |
| **Hasenclever-IPS** | | | | |
| Low/ Low-intermediate | 18 | 11 | 0.021 | 5.304 |
| High-intermediate/High | 2 | 10 |  |  |
| LTR, long term responders; prHL, primary refractory Hodgkin lymphoma; rHL, relapsed Hodgkin lymphoma | | | | |

**Supplementary Table 3.** Exemplary annotation of notable alterations in rHL/prHL samples to underline their biologic significance - see separate .xlsx file

**Supplementary Table 4.** All variants described by targeted hybrid capture sequencing - see separate .xlsx file

**Supplementary Table 5.** Number of significantly mutated genes showing positive selection and mean values of dn/ds ratios.

| **Group** | **Genes q < 0.1** | **Mean w** |
| --- | --- | --- |
| Responder | 85 | 185.0 |
| Primary refractory initial diagnosis | 25 | 443.7 |
| Primary refractory progression | 20 | 1,091.0 |
| Progression initial diagnosis | 65 | 345.6 |
| Progression relapse | 75 | 337.3 |

**Supplementary Table 6.** Summary of the occurrence of characteristic alterations over time in the samples studied.

**Supplementary Figures**

**
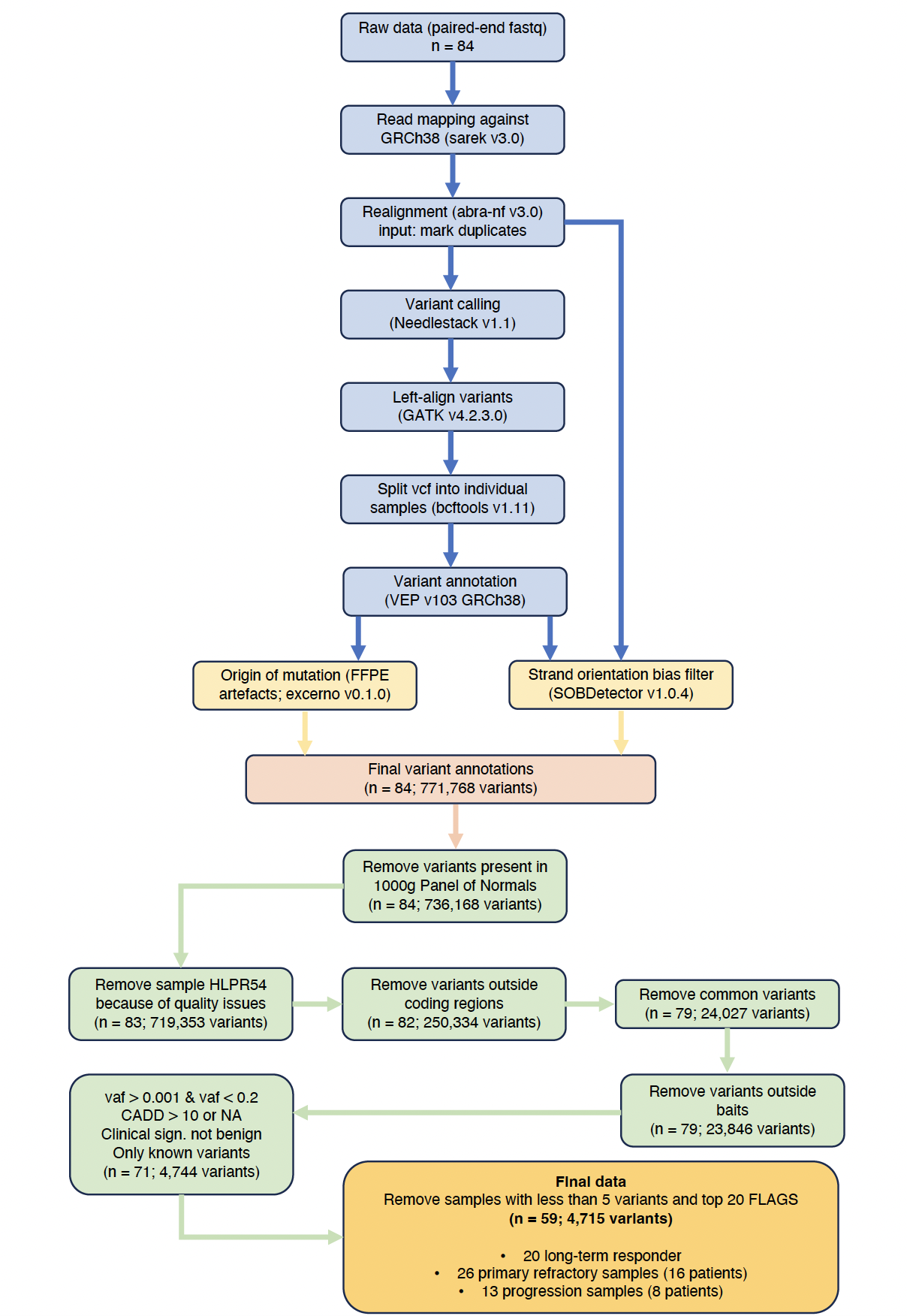
**

**Supplementary Figure 1.** Flow chart visualizing the processing workflow of variant filtering.

**Supplementary Figure 2.** 61-year-old female patient with rHL (case no. HL28). (1) Maximum intensity projections (MIP) of FDG PET, (2) axial fusion PET/CT imaging and (3) axial CT scan. (A) At initial staging, the patient had extensive disease stage IV with nodal involvement on both sides of the diaphragm, suspicious FDG-avid hepatic, spleen and kidney lesions. (B) Interim scanning after 2 cycles of chemotherapy (ABVD) showed good metabolic response; however, Deauville 4 lesions in the mediastinum and increased FDG uptake in the kidney lesion (red arrow) (Deauville 5). (C) After completion of 4 cycles of ABVD, there was persistent tumor burden in the left kidney and suspected relapse in the liver (yellow arrow). (D) PET imaging after treatment switch (1 cycle ICE + 1 cycle DHAP) revealed progressive disease in size and number of hepatic manifestations (yellow arrows) and persistent metabolic activity in the kidney. (E) PET/CT performed after one more cycle DHAP underscored progressive FDG-uptake in lymphoma lesions of the liver (yellow arrows) and left kidney (red arrow). Sufficient biopsy material was available from E (liver biopsy).

**Supplementary Figure 3.** 21-year-old male patient with rHL (case no. HL20). (1) Maximum intensity projections (MIP) of FDG PET, (2) axial fusion PET/MRI and (3) axial T2w MRI. (A) Initial PET image showed stage II disease with FDG-avid cervical, periclavicular and mediastinal lymph nodes and a possible affection of Waldeyer's ring. (B) PET/MRI after completion of 4 cycles of ABVD revealed decrease in size and intensity of FDG uptake at all lymph node sites of disease with the intensity of uptake below uptake in the liver; however suspicious new metabolic manifestation in a left axillary lymph node (red arrow). (C) relapse situation with increased FDG uptake in left sided cervical lymph nodes (yellow arrow). (D) After additional two cycles of chemotherapy PET/MRI showed residual disease in a cervical lymph node on the left side (yellow arrow) (Deauville 5). (E) after chemotherapy (DHAP + brentuximab + HD-CTx with BEAM) and stem cell transplantation PET imaging demonstrated complete metabolic response and physiological increased cervical muscle uptake. (F) Increased FDG uptake in histological proven lymphoma associated lesions of the scalp; moderate symmetrically increased FDG uptake in non-enlarged most likely inflammatory cervical lymph nodes. Sufficient biopsy material was available from A (cervical/supraclavicular nodal manifestation) and C (cervical/supraclavicular nodal manifestation).

**Supplementary Figure 4.** 20-year-old male with rHL (case no. HL10). (1) Maximum intensity projections (MIP) of FDG PET, (2) axial fusion PET/CT imaging and (3) axial CT scan. (A) Initial PET image revealed stage II disease with a large FDG-avid lymphoma mass (bulky disease) in the anterior mediastinum. (B) PET/CT after six cycles of chemotherapy (BEACOPP esc) showed significant decrease in size and intensity of FDG-avid tumor burden; however, there was residual metabolically active disease in the left anterior mediastinum (red arrow) (Deauville 4). (C) Increase of size of FDG-avid residual lymphoma with similar FDG uptake (red arrow) (Deauville 4). (D) After additional radiotherapy extensive relapse in mediastinal, right hilar lymph nodes and pulmonary manifestations occurred. (E) PET/CT after salvage chemotherapy and consecutive autologous stem cell transplantation (DHAP, stem cell apheresis, HD-CTx with BEAM) showed persistent FDG-avid lymphoma in right hilar lymph nodes und right lung. Sufficient biopsy material was available from C (mediastinal manifestation) and D (pulmonary manifestation via bronchoscopy).

**
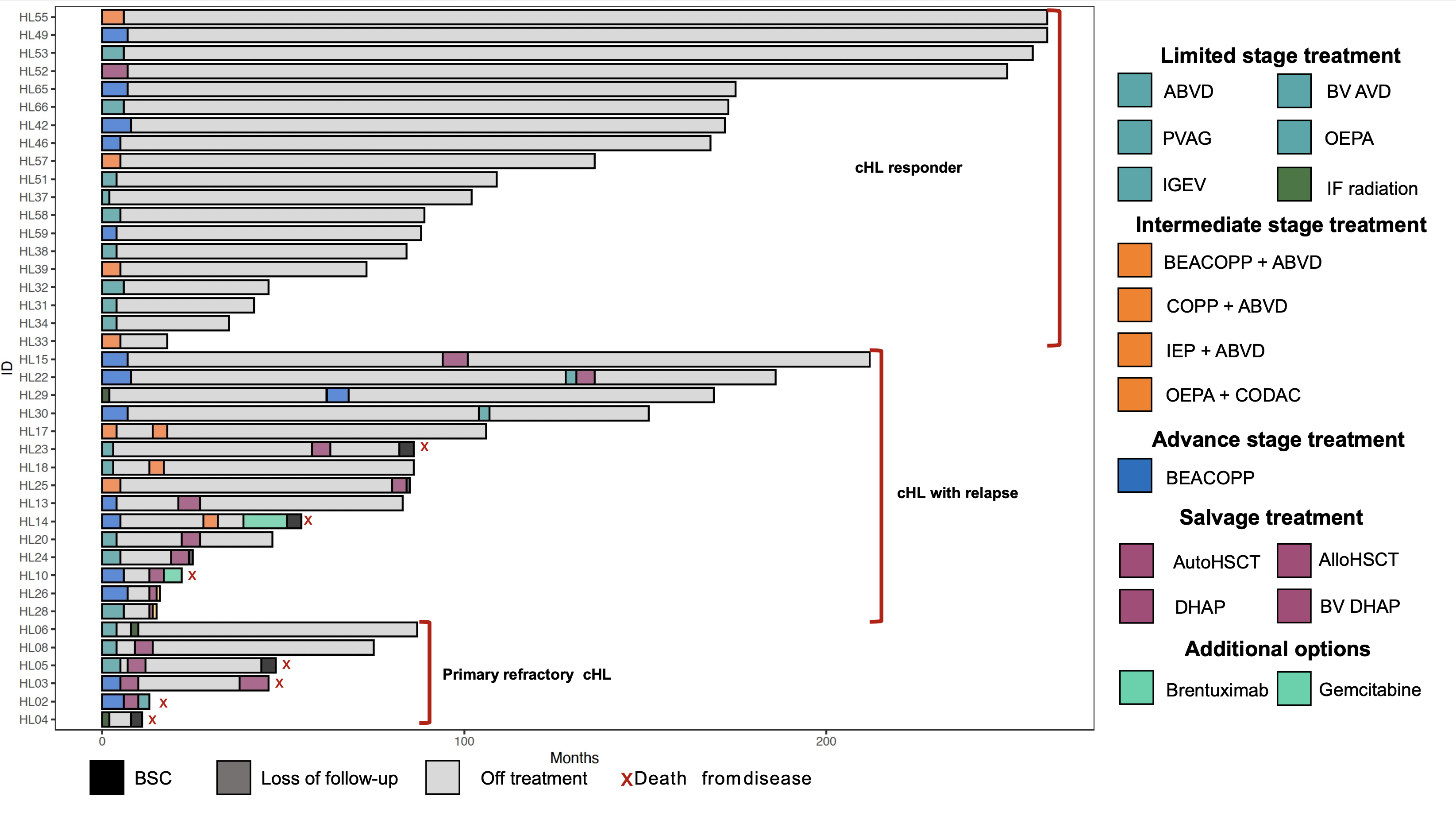
**

**Supplementary Figure 5.** Swimmer plot that illustrates the clinical course of responders, cHL cases experiencing relapse events (rHL) after initially responding towards standard polychemotherapy and primary refractory cHL (prHL).


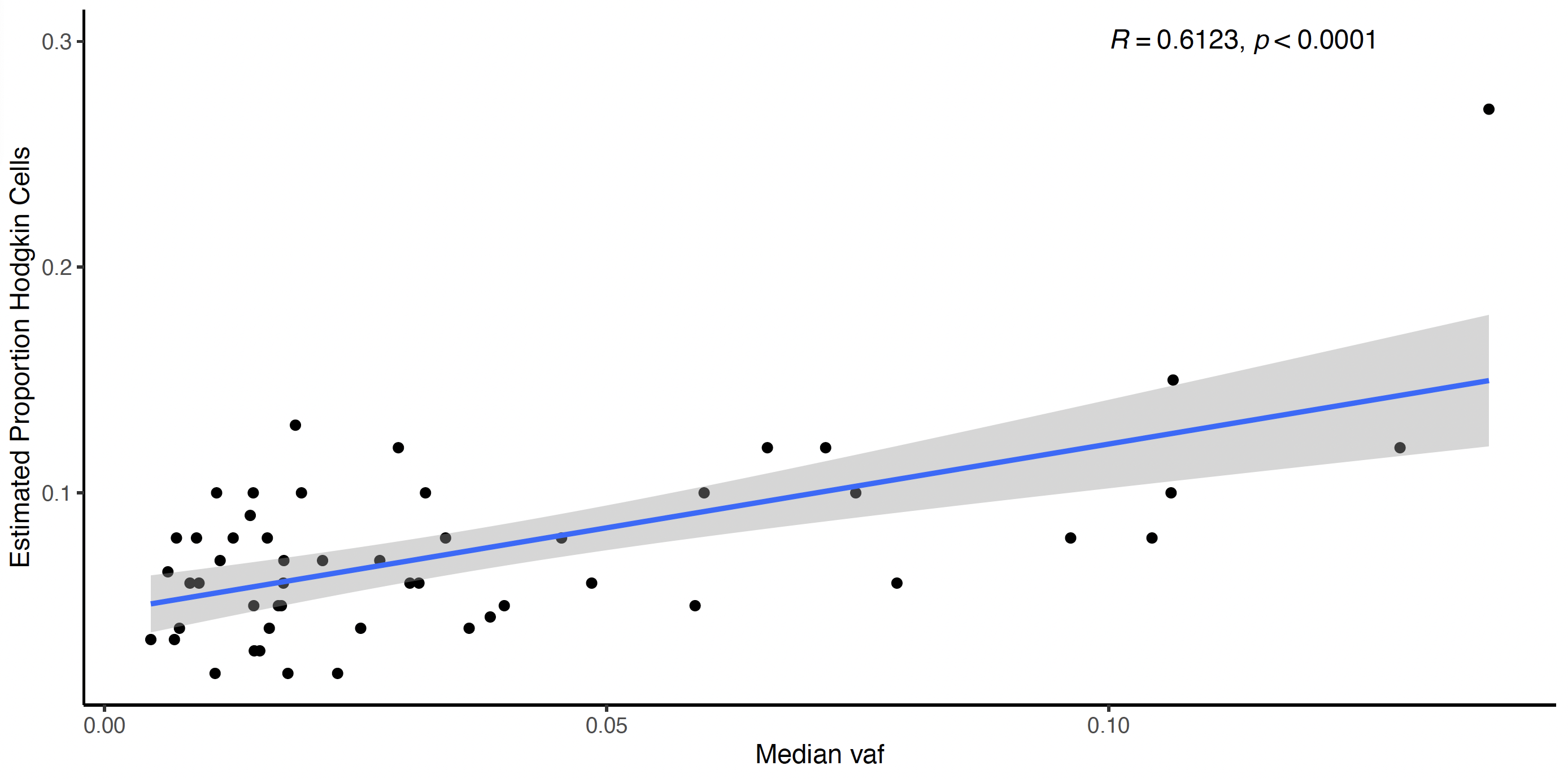


**Supplementary Figure 6.** Pearson correlation analysis, which assesses the relationship between the median VAF of each sample and the proportion of Hodgkin cells counted.

**
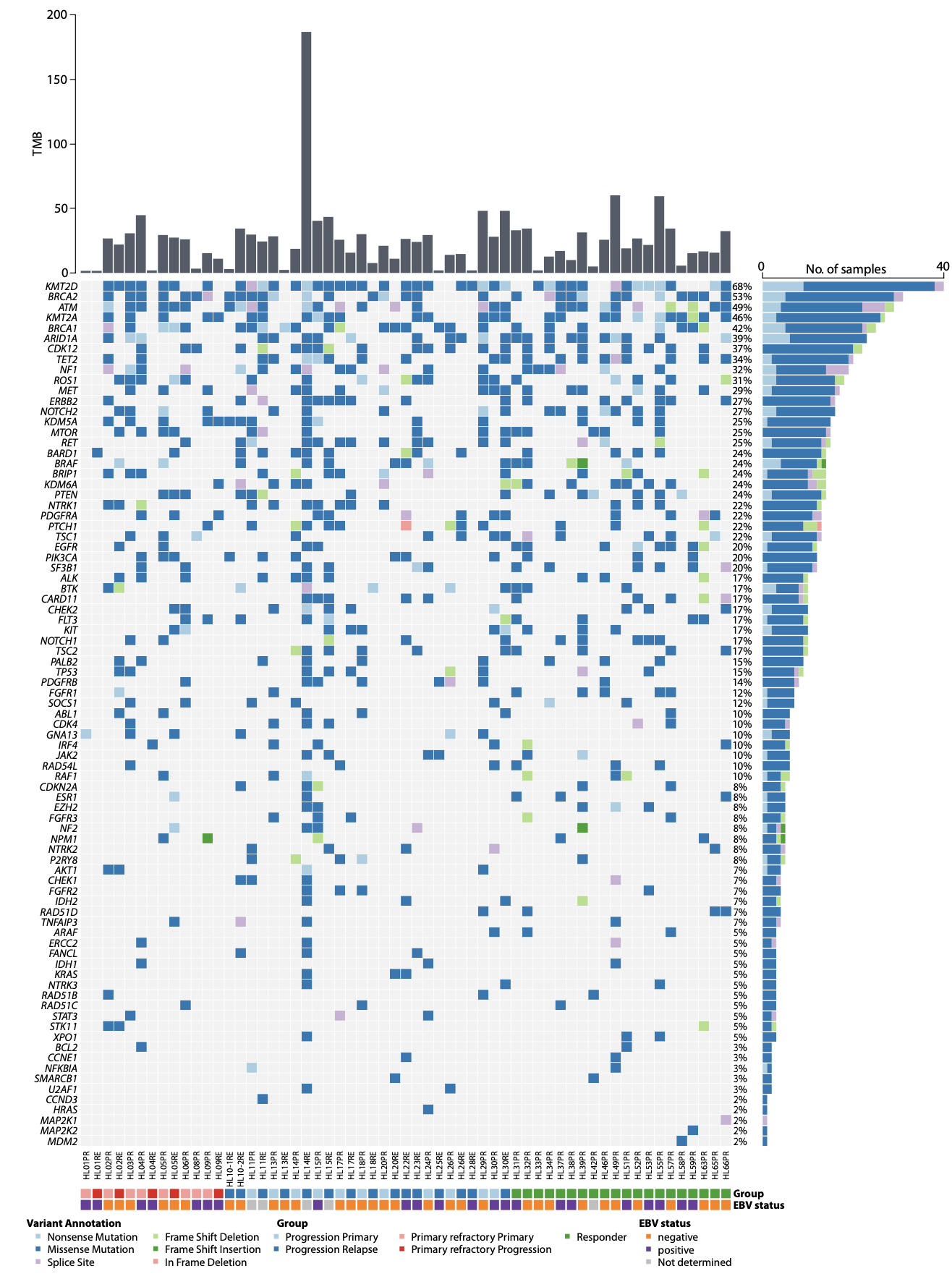
**

**Supplementary Figure 7.** Full Oncoplot visualizing the entire spectrum of somatic variants detected across all samples.

**
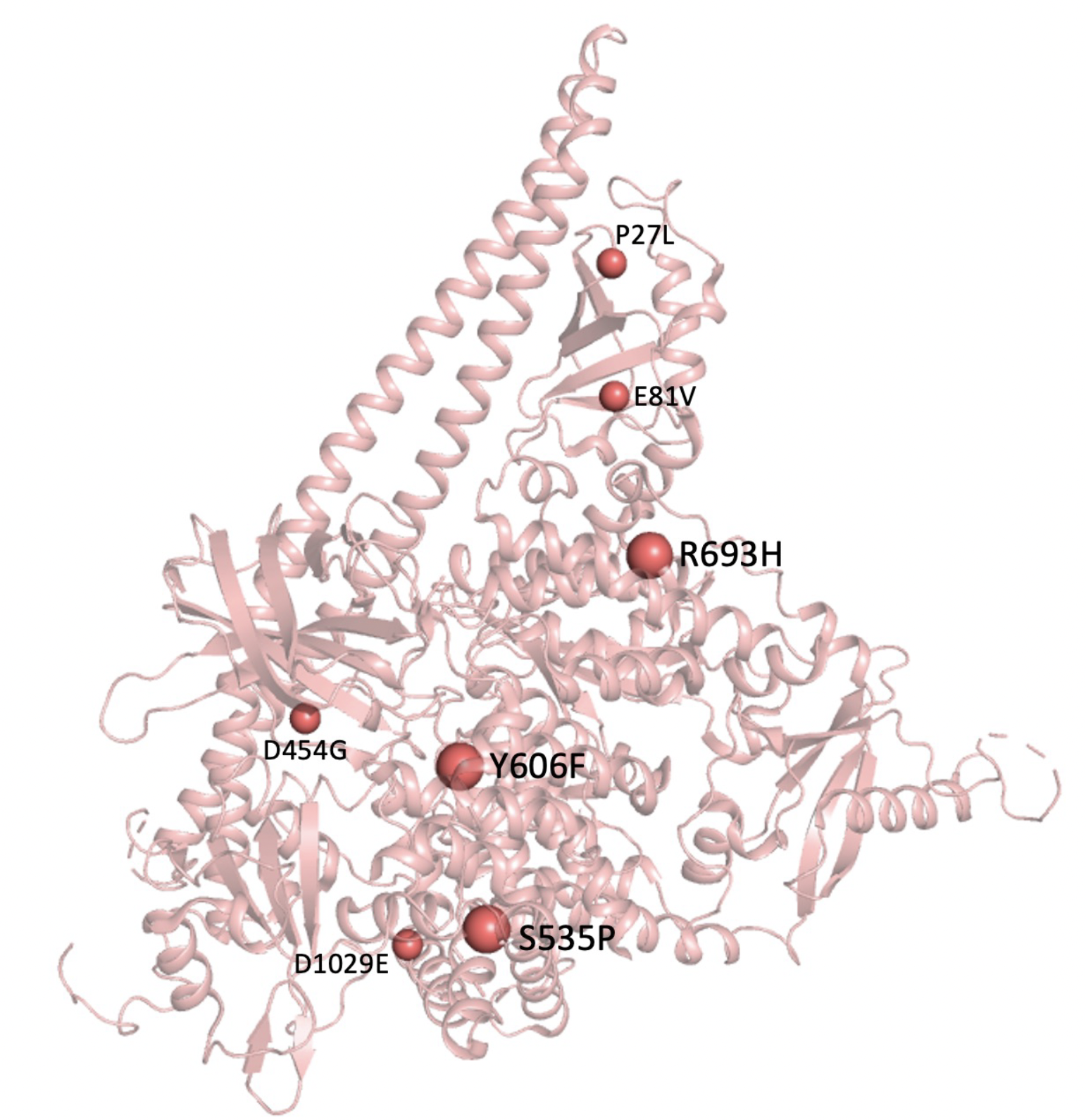
**

**Supplementary Figure 8.** 3D model of *PIK3CA* (PDB ID: 8DCP) shows the spectrum of missense variants (highlighted as spheres in dark salmon) found in the study group affecting exclusively gene sequences of functional relevance. **The image was generated using PyMol Schrödinger.**

**
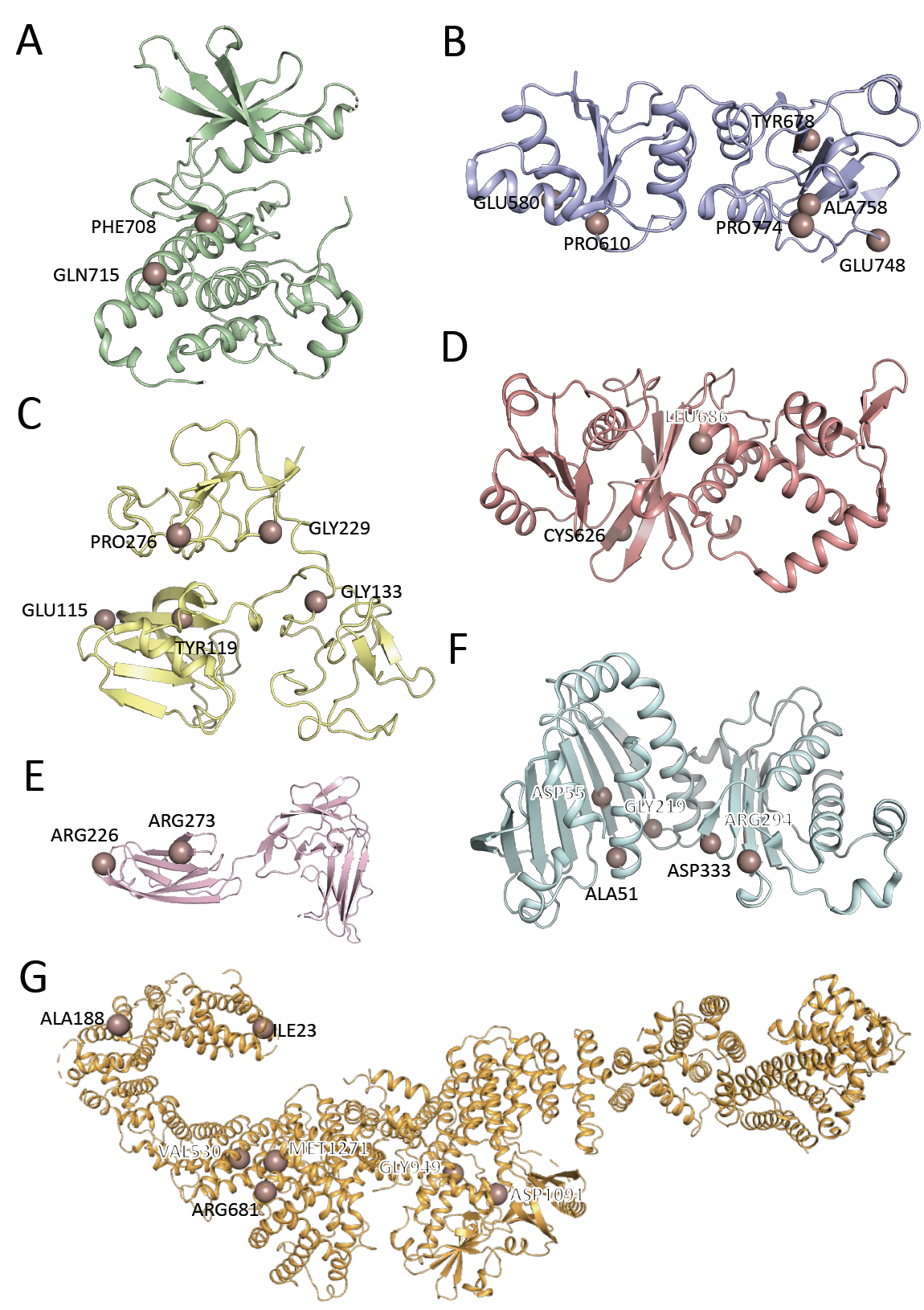
**

**Supplementary Figure 9.** Cartoon representation of structures of A) Human Ephb Tyrosine Kinase Domain (green, PDB: 3ZFX); B) BARD1 BRCT domain (purple, PDB: 2NTE); C) Human Hepatocyte Growth Factor (yellow, PDB: 3HN4); D) KAT6A (salmon, PDB: 8DD5); E) Platelet-derived growth factor subunit B (pink, PDB: 3MJG); F) PMS2 variant (cyan, PDB: 6MFQ), G) Isoform of Neufibromin I (orange, PDB: 7R03) highlighted with residues (spheres, brown) found as mutations in the respective gene and are found as mutations in the respective gene and are found to attenuate their respective functions.


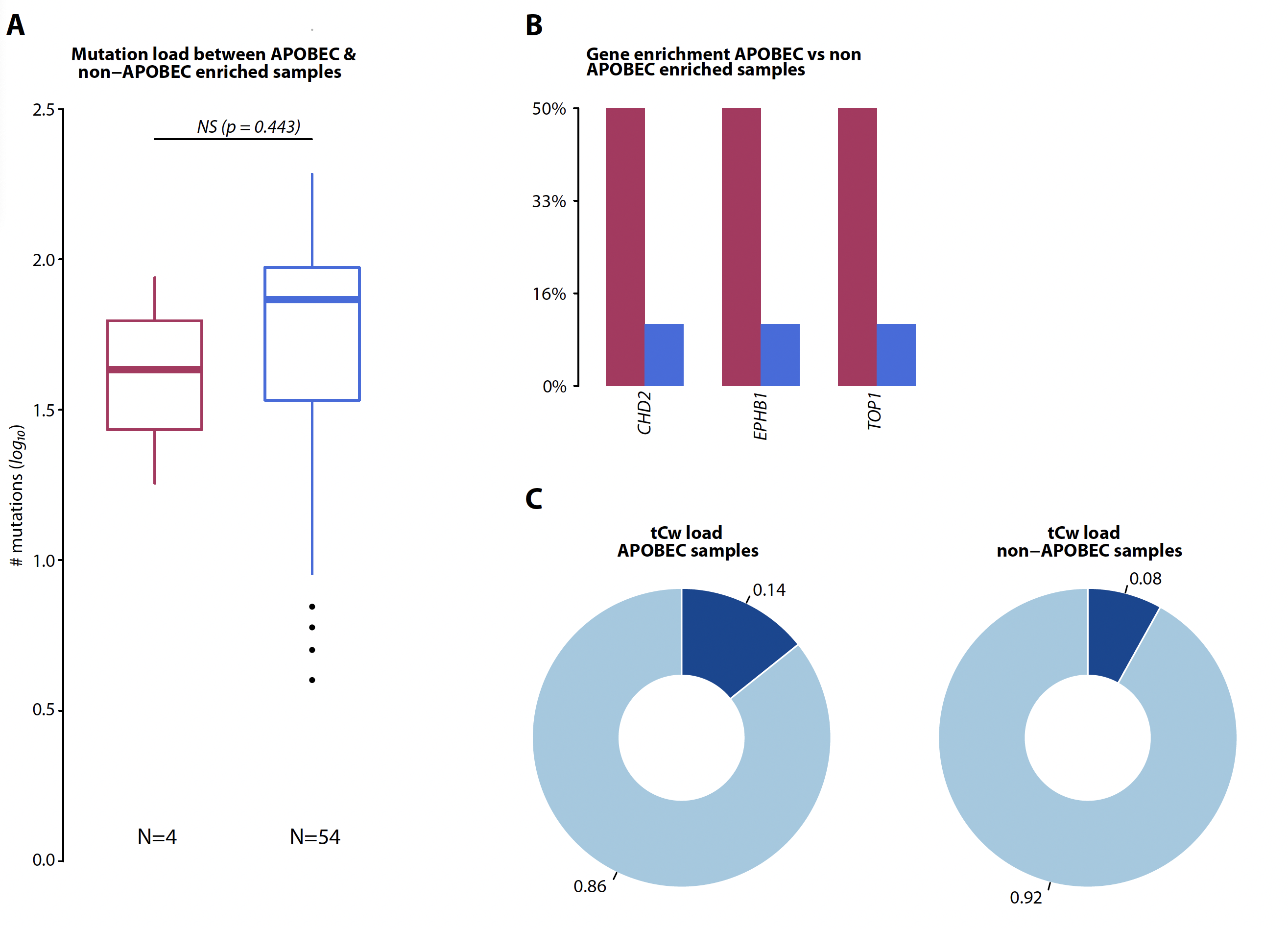


**Supplementary Figure 10.** APOBEC gene signatures among cHL cases included in our study. (A) Tumor mutational burden between APOBEC (red) and non-APOBEC enriched samples and (B) gene enrichment in APOBEC samples (p < 0.1). The distinct tCw motif distribution is shown as the proportion of the total number of observed mutations (dark blue refers to tCw load and light blue shows non tCw load).

**
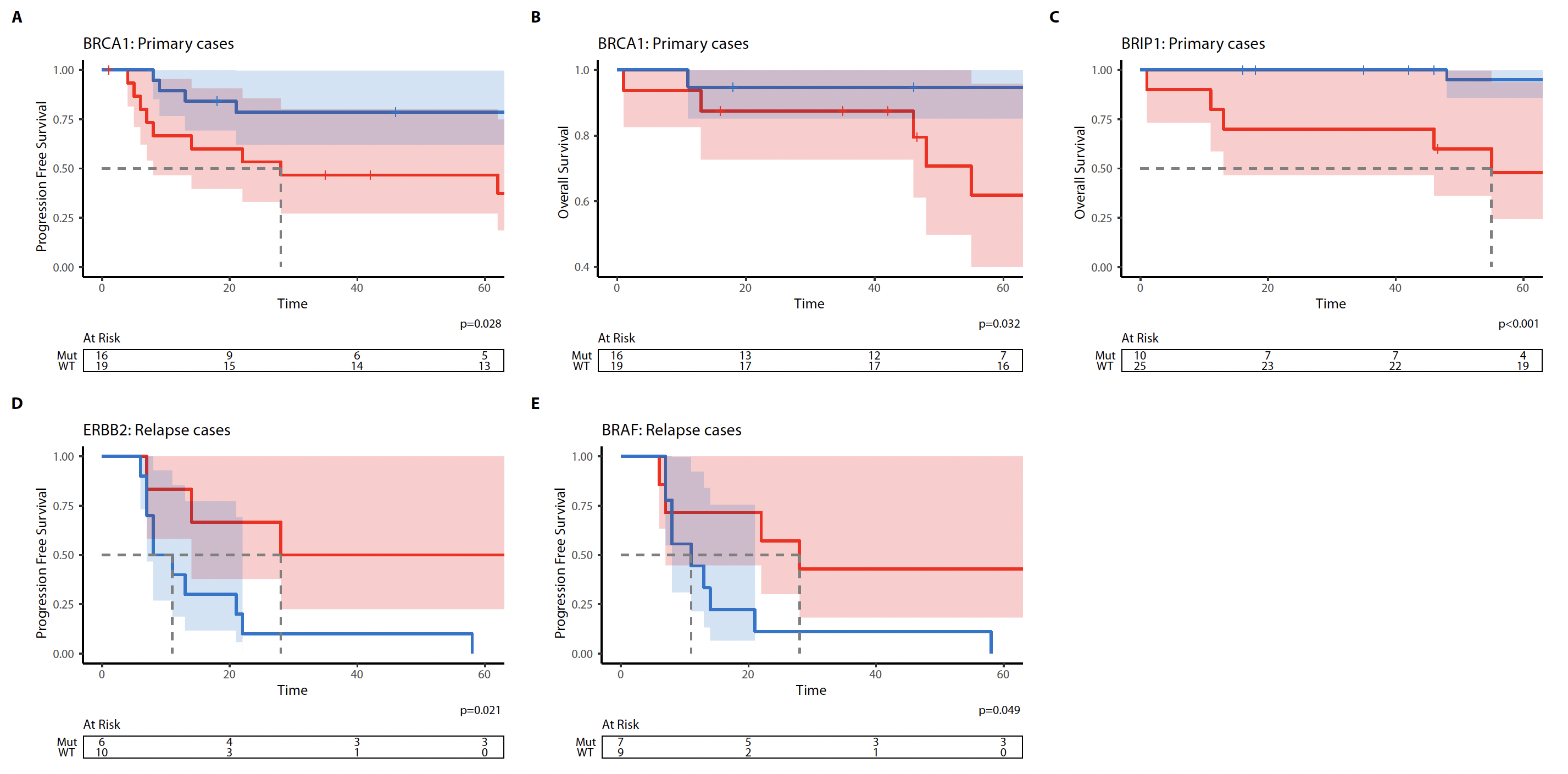
**

**Supplementary Figure 11.** Results from Kaplan Meier analysis for mutations (*BRCA1, BRIP1, ERBB2, BRAF*) with significant impact on either PFS (A, D, E) or OS (B, C). Mutated samples are denoted by red color coding and wild-type samples by blue.


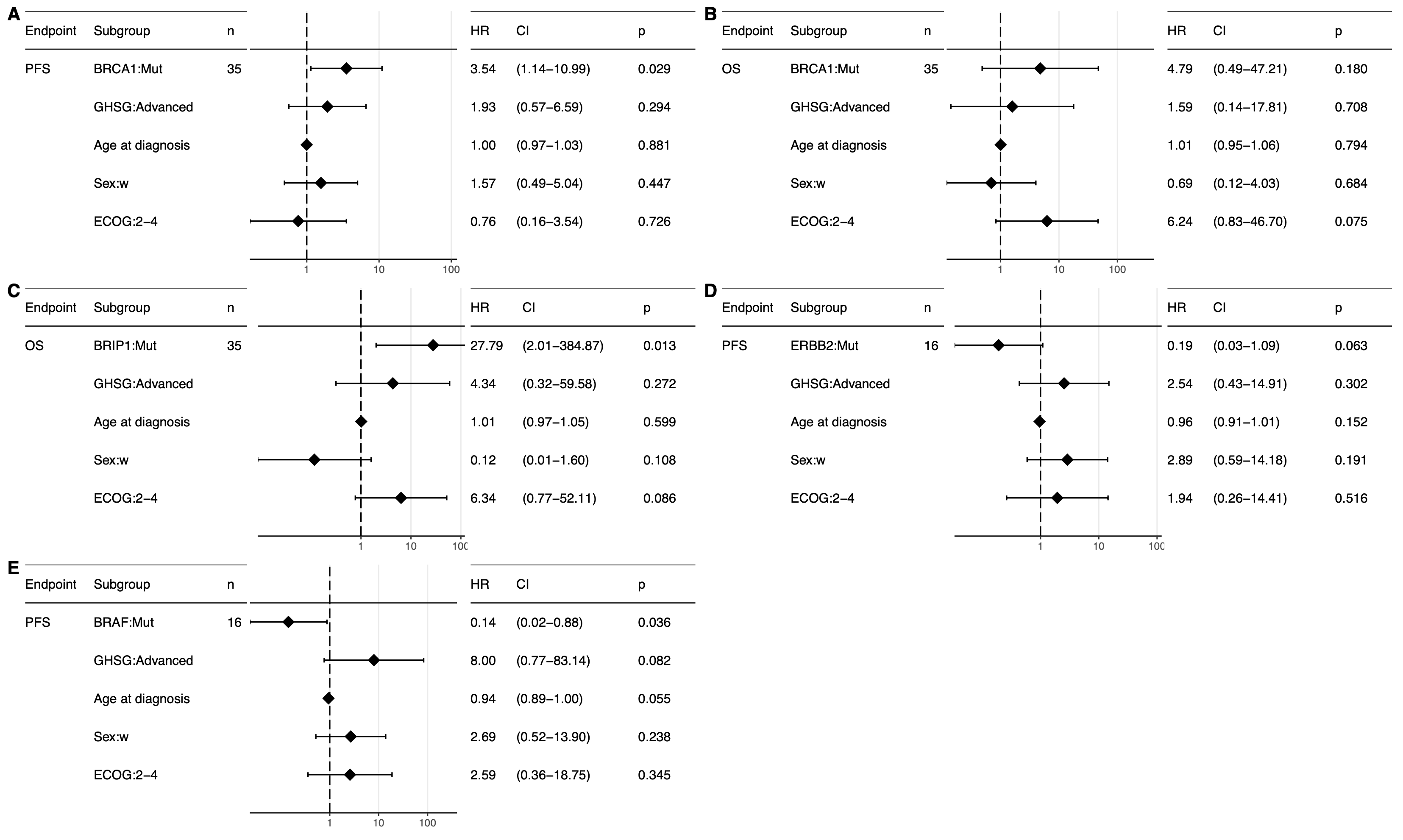


**Supplementary Figure 12.** Multivariate analysis using a Cox proportional hazard model to investigate the independence of the prognostic value of molecular markers identified as prognostically relevant in the Kaplan Meier analysis. The multivariate analysis included clinical parameters that are established as independent risk factors to predict survival of cHL patients. The plots show the hazard ratio and the 95% confidence interval.

**Supplementary References**

1. Swerdlow SH, Campo E, Pileri SA, et al. The 2016 revision of the World Health Organization classification of lymphoid neoplasms. *Blood*. 2016;127(20):2375-2390.

2. Kwee TC, Kwee RM, Nievelstein RA. Imaging in staging of malignant lymphoma: a systematic review. *Blood*. 2008;111(2):504-516.

3. Ewels PA, Peltzer A, Fillinger S, et al. The nf-core framework for community-curated bioinformatics pipelines. *Nat Biotechnol*. 2020;38(3):276-278.

4. Garcia M, Juhos S, Larsson M, et al. Sarek: A portable workflow for whole-genome sequencing analysis of germline and somatic variants. *F1000Res*. 2020;9:63.

5. Mose LE, Perou CM, Parker JS. Improved indel detection in DNA and RNA via realignment with ABRA2. *Bioinformatics*. 2019;35(17):2966-2973.

6. Delhomme TM, Avogbe PH, Gabriel AAG, et al. Needlestack: an ultra-sensitive variant caller for multi-sample next generation sequencing data. *NAR Genom Bioinform*. 2020;2(2):lqaa021.

7. McKenna A, Hanna M, Banks E, et al. The Genome Analysis Toolkit: a MapReduce framework for analyzing next-generation DNA sequencing data. *Genome Res*. 2010;20(9):1297-1303.

8. McLaren W, Gil L, Hunt SE, et al. The Ensembl Variant Effect Predictor. *Genome Biol*. 2016;17(1):122.

9. Rentzsch P, Witten D, Cooper GM, Shendure J, Kircher M. CADD: predicting the deleteriousness of variants throughout the human genome. *Nucleic Acids Res*. 2019;47(D1):D886-D894.

10. Kandoth C GJ, Mattioni M, Struck A, Boursin Y, Penson A, Chavan S. mskcc/vcf2maf: vcf2maf v1.6.16 (v1.6.16). Zenodo. ; 2018.

11. Diossy M, Sztupinszki Z, Krzystanek M, et al. Strand Orientation Bias Detector to determine the probability of FFPE sequencing artifacts. *Brief Bioinform*. 2021;22(6).

12. Mitchell A, Ruiz M, Yang S, Wang C, Davila JI. Excerno: Using Mutational Signatures in Sequencing Data to Filter False Variants Caused by Clinical Archival. *J Comput Biol*. 2023;30(4):366-375.
